# Feasibility and Safety of Endovascular Aneurysm Repair (EVAR) in a District General Hospital: A Retrospective Study of 10-Year Outcomes

**DOI:** 10.7759/cureus.72669

**Published:** 2024-10-29

**Authors:** Dani Avabde, Mohammad Mostafizur Rahman Miah, Walid Alnatsheh, Peter Lee Chong, Asghar Butt

**Affiliations:** 1 Vascular Surgery, Nottingham University Hospitals NHS Trust, Nottingham, GBR; 2 Vascular Surgery, Manchester Royal Infirmary, Manchester, GBR; 3 Vascular Surgery, United Lincolnshire Hospitals NHS Trust, Boston, GBR

**Keywords:** abdominal aortic aneurysm (aaa), district general hospital, endovascular aneurysm repair (evar), feasibility and safety, stent endoleak

## Abstract

Background: Endovascular aneurysm repair (EVAR) represents a pivotal advancement in the treatment of abdominal aortic aneurysm (AAA).

Objective: The primary objective of this study is to present a comprehensive overview of the EVAR service in a district-level vascular centre and to compare it with the national outcome to outline a service model for other district general hospitals.

Method: Patients who underwent an EVAR procedure from 2015 over seven years were included. Retrospective data were collected from post-EVAR surveillance, and the National Vascular Registry (NVR) database was included.

Result: A total of 99 patients were included. Most of the patients (96%) who underwent EVAR were over 65 years old at the time of the procedure and predominantly male, accounting for 90%. The 30-day post-EVAR mortality rate was low (0.9%). Most of the patients had no primary endoleaks (80%), which was almost comparable to the national standard (82.5%).

Conclusion: This study showed the feasibility of successful implementation and safety of the EVAR program in a small district-level hospital. The outcome was comparable to the national standard. This study has identified the strengths and areas for improvement in the EVAR programme in such a setting.

## Introduction

An abdominal aortic aneurysm (AAA) is defined as an enlargement of the abdominal aorta exceeding the normal vessel diameter by more than 50%. AAA affects 4% of males in the UK, with smoking, hypertension, and age as major risk factors [1]. Early detection and intervention reduce morbidity and mortality. EVAR has transformed AAA management, offering a minimally invasive alternative to open surgery with reduced perioperative risks and shorter recovery times. The natural progression of AAA involves continuous aneurysm growth and increased risk of rupture, with the probability of both increasing with aneurysm size. Ruptured AAAs have a mortality rate exceeding 50%, making early detection and intervention crucial for reducing AAA-related morbidity and mortality. For aneurysms measuring between 5.5 cm and 6.9 cm, the annual rupture risk is approximately 9%, increasing to over 32% for aneurysms larger than 7 cm [2]. Despite a decline in age-standardized death rates from AAA in Europe and the USA over the past 30 years, AAA remains a significant cause of mortality, accounting for five and two deaths per 100,000 in men and women, respectively [3]. This decline is attributed to various factors, including behavioural changes, such as reduced tobacco smoking, earlier detection through national AAA screening programs in the UK, Sweden, and the USA, and advancements in medical treatment.

Management of AAA primarily involves open surgical repair (OSR) and endovascular aneurysm repair (EVAR). OSR, though effective, is a major abdominal surgery associated with substantial perioperative risks and prolonged recovery periods. In contrast, EVAR offers a minimally invasive alternative, utilizing stent grafts deployed from within the aorta through remote arterial access to exclude the aneurysm. Despite its benefits, EVAR patients require regular clinical and imaging follow-ups to monitor for complications such as endoleaks, which can lead to aneurysm growth and rupture. These complications arise from various mechanisms, including retrograde blood flow from aortic branches and loss of graft component seals. Revision endovascular procedures are often necessary to address these issues.

EVAR represents a pivotal advancement in the treatment of AAA, a condition characterized by the pathological dilation of the abdominal aorta, which poses a significant risk of rupture and subsequent mortality. Conventional OSR, while effective, is associated with substantial perioperative morbidity and prolonged recovery periods due to its invasive nature [4]. In contrast, EVAR offers a minimally invasive alternative, significantly reducing perioperative complications and facilitating expedited recovery [5]. The significance of EVAR in AAA management is profound. It extends therapeutic options to a broader cohort of patients, including those at high surgical risk who may not be candidates for traditional open repair. According to the National Vascular Registry (NVR) in the UK, the in-hospital postoperative mortality rate for EVAR is approximately 0.4%, compared to 3.3% for OSR. Additionally, EVAR is associated with shorter hospital stays (median of three days versus seven days for OSR) and quicker resumption of normal activities, with an average return to daily functions within two to three weeks compared to six to eight weeks for OSR [6].

The NVR's 2022 Annual Report highlights that approximately 4,000 EVAR procedures are performed annually in the UK, reflecting a steady increase in the adoption of this technique over the past decade [7]. This trend is driven by the growing body of evidence supporting the advantages of EVAR, including a lower incidence of major adverse cardiovascular events (MACE) and reduced need for secondary interventions compared to OSR. By providing a less invasive yet effective treatment modality, EVAR significantly improves the quality of life and survival outcomes for patients afflicted with this potentially life-threatening condition. Its critical role in modern vascular surgery is underscored by its ability to deliver superior clinical outcomes, enhance patient recovery, and reduce healthcare costs through shorter hospital stays and fewer complications.

This study evaluates the feasibility and safety of implementing EVAR service in a district general hospital. By analysing outcomes from November 2015 to March 2022, we assess whether smaller hospitals can achieve standards comparable to national benchmarks. The findings highlight key factors for successful service delivery and strategies for optimising patient outcomes, supporting the case for expanding EVAR services at the district level in the UK.

## Materials and methods

Patients who underwent an EVAR procedure at our institution between November 16, 2015, and March 31, 2022, were included. Patients were only included if they had complete data from post-EVAR surveillance and the NVR database. Patients who underwent open surgical repair for AAA were excluded. Patients with incomplete or missing data in the theatre logbook and associated records and patients who were lost to follow-up prior to their post-EVAR surveillance appointments were also excluded.

Patient details, including demographics, graft types, and dates of surgery, were documented contemporaneously in theatre records and subsequently transferred to an Excel spreadsheet. Additional data, including operative notes (available primarily after 2017) and booking forms, were located and reviewed to confirm the type of surgery and timing. Patient letters, discharge summaries, and follow-up notes were retrieved from the hospital database for comprehensive data collection. Details from post-EVAR surveillance clinics, including follow-up imaging and clinical assessments, were incorporated into the dataset. Relevant data from the NVR database were included to ensure completeness and accuracy.

Primary outcomes included 30-day mortality, incidence of primary and secondary endoleaks, complication rates, and length of hospital stay. Endoleaks were classified according to standard definitions (types I, II, III, etc.). Descriptive statistics were used to summarize patient demographics and outcomes. Continuous variables were expressed as means ± standard deviations or medians with interquartile ranges, depending on the data distribution. Categorical variables were presented as frequencies and percentages. Comparisons with national data were made using chi-square tests for categorical variables and t-tests for continuous variables, with a significance level set at p < 0.05.

In our hospital, there is a follow-up protocol for the EVAR patients after surgery. EVAR patients undergo a duplex ultrasound scan and an abdominal X-ray (anterior-posterior and lateral views) on postoperative day one (POD1). This is to look for endoleaks and check the position of the stent. All patients would then have a CT angiogram of the aorta at three months from the operation date. Follow-up is then yearly thereafter.

In this retrospective cohort study, descriptive datasets were presented in the form of mean and proportion. The analysis was performed in three steps. A series of univariate analyses was performed using different datasets. Then, multivariate analysis was performed using these results. Finally, a stepwise model was performed. The ultimate goal was to enhance clinical decision-making processes and improve patient care by understanding the relative benefits and risks associated with EVAR in the context of the district general hospital setup.

The collected data were subjected to a retrospective review, with a focus on key outcome measures, such as procedural success, complication rates, reintervention rates, and patient survival. The data were organised and managed using Excel for preliminary analysis and subsequently imported into statistical software for detailed analysis. Descriptive statistics were calculated to summarise patient demographics, procedural details, and outcome measures. Comparative analysis against national benchmarks was conducted to evaluate our performance relative to established standards. This included comparing our complication rates, reintervention rates, and overall patient outcomes with those reported in national registries and published literature.

This retrospective study was conducted in accordance with ethical standards and institutional review board guidelines and was registered and approved by the appropriate hospital body. Patient confidentiality was maintained, and all data were anonymised prior to analysis.

## Results

**Figure 1:**
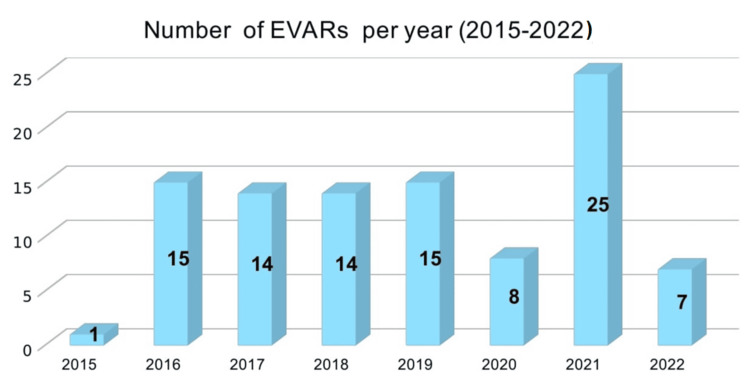
Bar graph showing the number of EVARs performed per year (2015-2022). EVAR - endovascular aneurysm repair

The annual number of EVAR procedures performed at our institution from 2015 to March 2022 reflects the evolving landscape of AAA management within our facility. A total of 100 patients underwent EVAR during this period; however, one patient was excluded due to incomplete data. Thus, a total of 99 (n=99) patients were included. From 2016 to 2019, the number of EVAR cases remained relatively stable, averaging around 15 procedures per year (Figure 1). This consistency indicates a steady adoption and application of the EVAR technique in treating AAA. However, in 2020, there was a significant drop in the number of EVAR procedures, with only eight cases performed. This decline can be attributed to the COVID-19 pandemic, which disrupted elective surgical schedules and healthcare operations globally.

In contrast, 2021 saw a marked increase in the number of EVAR procedures, rising to 25 cases. This surge was primarily due to addressing the backlog of cases from the previous year, as healthcare services resumed more regular operations. The fluctuation in annual EVAR procedures underscores the impact of external factors such as the pandemic on surgical volumes and highlights the institution's capacity to adapt and manage procedural backlogs effectively.

In our cohort of patients undergoing EVAR, the majority presented asymptomatically. Specifically, 90 patients (88.23%) were asymptomatic at the time of diagnosis and were subsequently scheduled for elective EVAR procedures. This high percentage of asymptomatic cases highlights the importance of routine screening and surveillance in the early detection of AAA, allowing for timely elective intervention. Conversely, 12 patients (11.76%) were symptomatic, presenting with clinical signs suggestive of AAA, necessitating a more urgent approach to their management. Additionally, two patients (1.98%) required emergency EVAR due to acute presentations, underscoring the critical nature of timely diagnosis and intervention in preventing rupture and associated mortality. These data reflect the spectrum of clinical presentations in AAA and the corresponding urgency of surgical intervention based on symptomatology.

In our study cohort, the vast majority of the patients (96%) who underwent EVAR were over 65 years old at the time of the procedure, with an age range spanning from 55 to 89 years, with a mean age of 72.5 years (Table 1). This underscores the higher prevalence of AAA among the elderly population. The gender distribution was predominantly male, accounting for 90% (n=89) of the patients, which is consistent with the known epidemiology of AAA. Additionally, a significant proportion of the patients had a history of hypertension (n=69, 70%). Other comorbidities include ischaemic heart disease (34.3%), diabetes mellitus (22.2%), renal disease (18.1%), and respiratory disease (21.2%) (Table 1). The majority of the patients were either current or former smokers (n=82, 83%). These findings highlight the common risk factors associated with AAA, including advanced age, male gender, hypertension, and smoking, which are critical considerations in the clinical management and prevention strategies for this condition.

**Table 1:** Demographics and risk factors.

Age	Minimum age: 55 years, maximum age: 89 years	Mean age: 72.5 years
Sex	Male: 90% (n=89), Female: 9.9% (n=10)	
Comorbidities/Risk factors	Hypertension	70% (n=69)
	Ischaemic heart disease	34.3% (n=34)
	Diabetes mellitus	22.2% (n=22)
	Renal disease	18.1% (n=18)
	Respiratory disease	21.2% (n=21)
	Current or ex-smoker	83% (n=82)

Post-procedural surveillance is critical for detecting endoleaks following EVAR. Primary endoleaks are identified in the first available surveillance scan before patient discharge, while secondary endoleaks are detected in subsequent scans in patients who had no initial evidence of endoleaks.

**Figure 2:**
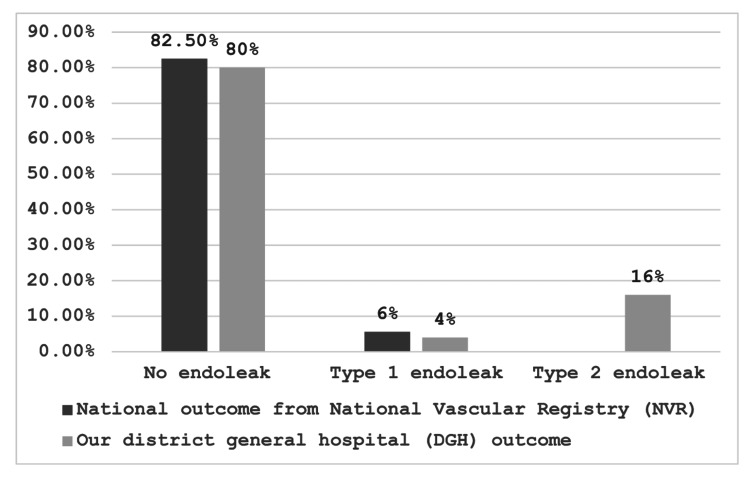
Comparison of outcomes regarding endoleak with the national standard.

In our institution, several primary endoleaks were noted post-EVAR. The vast majority of the patients had no primary endoleaks (80%), which was almost comparable to the national standard (82.5%) [7]. Type 1 endoleak was found in 4% of patients where it was slightly higher (5.6%) nationally (Figure 2) [7]. One patient, operated on in September 2016, initially suspected of having a type 1 (T1) endoleak, was later found to have a good proximal seal, suggesting a possible type 3 (T3) endoleak. Another case involved a patient initially suspected of a T1 endoleak, but further review with a university hospital revealed no T1 endoleak, with the issue likely being a type 2 (T2) endoleak from accessory renal vessels. Another patient had a T1 endoleak noted postoperatively, and after a discussion with a university hospital, a cuff extension and/or stent procedure was performed. Duplex scans identified a T1b endoleak in another patient, with CT angiography (CTA) showing an occluded left common femoral artery (CFA) that was managed conservatively. Additionally, a T1a endoleak was detected via duplex scan in another patient, leading to plans for a cuff extension with endo anchors at the tertiary referral hospital. Finally, an initial duplex scan detected a T2 endoleak in one patient, with a follow-up CTA indicating a T1a/b endoleak. This was likely a T2 endoleak, and the patient continued under surveillance to monitor sac size.

Secondary endoleaks were identified in six patients (5.94%) who initially showed no evidence of endoleaks in their first post-procedural scan but were later detected in follow-up imaging. All secondary endoleaks were classified as type 2, typically resulting from retrograde blood flow into the aneurysm sac from collateral arteries such as the lumbar or inferior mesenteric arteries. These findings underscore the necessity for ongoing, rigorous follow-up imaging to detect and manage endoleaks, ensuring the long-term success and safety of the EVAR procedure.

**Figure 3:**
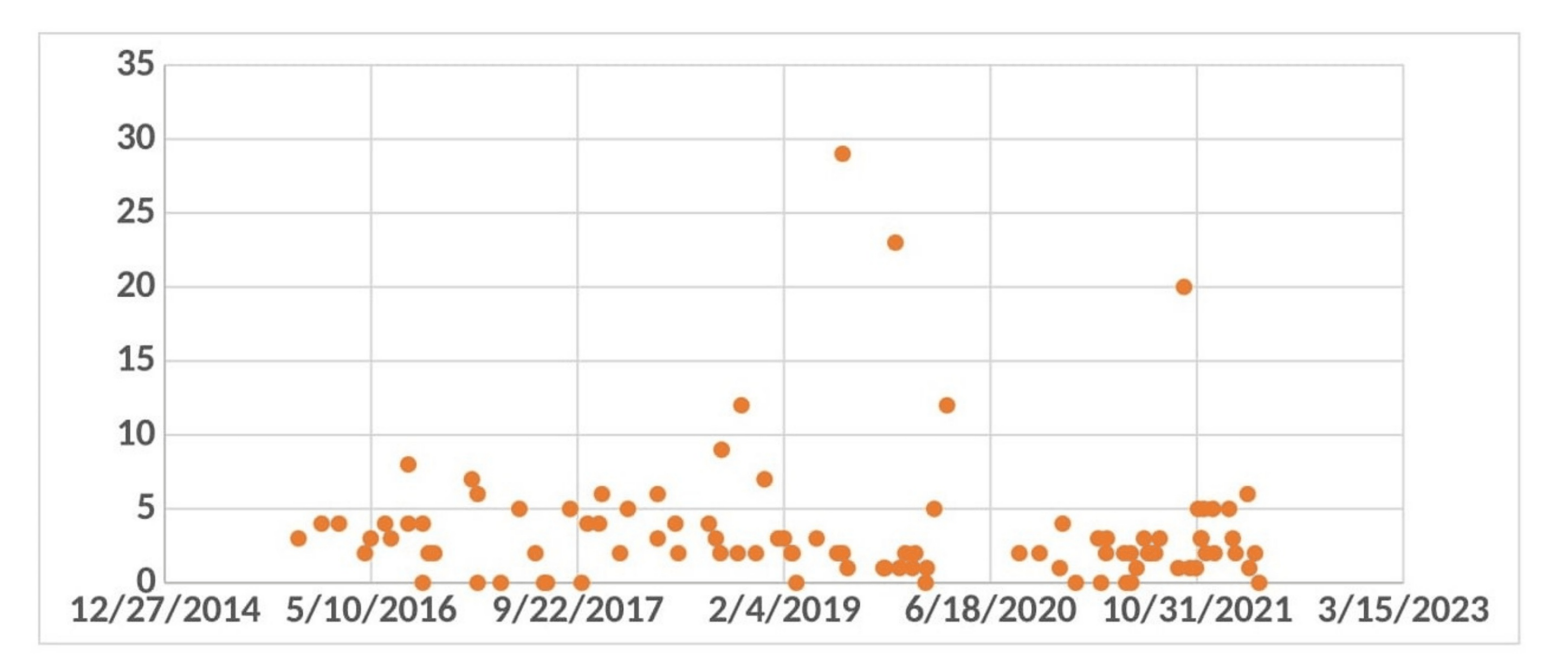
Overall postoperative hospital stay ranging from one night and max hospital stay of 29 days.

In 2020, the mean hospital stay for patients undergoing EVAR was 4.21 days, with a median stay of three days (Figure 3). Three patients experienced significantly prolonged hospitalizations of 20, 23, and 29 days, respectively. One patient's extended stay was due to an urgent admission for a per rectal (PR) bleed and an incidental AAA, followed by a post-EVAR chest infection that prolonged recovery. Another patient, who had an incidental finding of AAA on a CT scan and was urgently transferred to the vascular unit, was discharged two days post-EVAR, resulting in a total hospital stay of 23 days. A third patient, suspected of having a type 1 vs type 2 endoleak, required further investigations for diagnosis, resulting in a total stay of 20 days.

**Figure 4:**
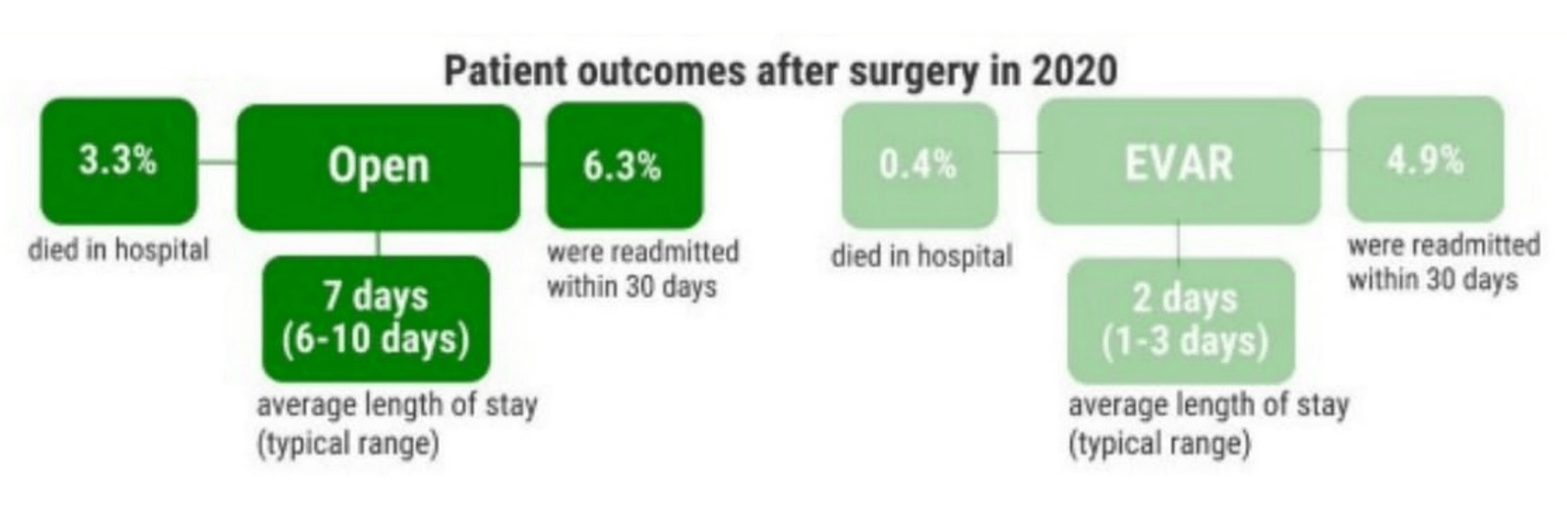
Comparing postoperative outcomes between open aneurysm repair and EVAR in 2020. EVAR - endovascular aneurysm repair

In 2021, the median hospital stay for 27 patients undergoing EVAR was reduced to two days, with a mean stay of 3.11 days (Figure 4). Only one patient had a prolonged stay of 20 days. The maximum hospital stay for three patients was five days each. These findings suggest improvements in perioperative care and post-operative management, likely contributing to shorter hospital stays and quicker recoveries for most patients. The data also highlight the variability in hospital stay durations due to individual patient complications and the need for tailored post-operative care.

In our cohort, the 30-day EVAR mortality rate was 0.9%, with one recorded death occurring in 2016. This patient passed away 26 days following the procedure. Unfortunately, no electronic discharge document was available online to provide further details about the cause of death. This incident underscores the critical importance of thorough post-operative monitoring and documentation to better understand and mitigate potential complications associated with EVAR. Despite the limited data on this particular case, the overall low mortality rate suggests that EVAR is generally a safe procedure within the first 30 days post-operation in our institution. However, continuous efforts are needed to ensure comprehensive follow-up and detailed record-keeping to improve patient outcomes further.

The post-operative morbidity following EVAR at our institution was monitored from electronic discharge documents up to the first consultant review, approximately six weeks after the procedure. During this period, complications were observed in a subset of patients, highlighting the range of potential morbidities associated with EVAR. Specifically, three patients developed chest infections, which required medical intervention and prolonged recovery times. Additionally, two patients experienced wound infections, necessitating further treatment to prevent more severe complications. One patient developed a seroma, a localised accumulation of clear fluid that sometimes occurs post-surgery, which needed to be managed to ensure proper healing. These findings emphasize the importance of vigilant post-operative care and monitoring to promptly identify and treat complications, thereby improving overall patient outcomes and recovery after EVAR.

During the study period, a variety of endovascular devices were utilized for EVAR procedures at our institution. The most frequently used device was the Cook Medical stent graft, employed in 69 cases, representing 63.9% of all procedures. The Cordis device was the second most commonly used, with 23 cases, accounting for 21.3% of the total. The Gore device was used in seven cases, making up 6.5% of the procedures. Lastly, the Anaconda device was utilized in four cases, which is 3.7% of the total EVAR procedures. This distribution reflects the preferences and availability of different devices, as well as the specific anatomical and clinical considerations that influence device selection in EVAR. The predominance of Cook Medical devices indicates a preference or suitability for this particular stent graft in the majority of cases managed at our institution.

## Discussion

Our institution's experience with EVAR from 2015 to March 2022 highlights both the consistency and adaptability of our approach to AAA management. Annually, the number of EVAR procedures averaged around 15 from 2016 to 2019, reflecting a steady adoption rate. However, the COVID-19 pandemic in 2020 caused a significant drop to only eight cases due to disrupted elective surgeries. In 2021, we saw a rebound with 25 procedures as we addressed the backlog, illustrating our capacity to adapt to external challenges. Nationally, the NVR also reported similar trends, with fluctuations influenced by the pandemic but generally maintaining a high volume of EVAR procedures across the UK.

The clinical presentation of our patients shows that 88.23% were asymptomatic at diagnosis, aligning with national data emphasizing the importance of routine screening for early detection. In contrast, 11.76% were symptomatic, and 1.98% required emergency EVAR, underscoring the varied clinical presentations and the critical need for timely interventions.

Demographically, our patient cohort was predominantly elderly, with 96% over 65 years old, and predominantly male (90%), which is consistent with national demographics reported by the NVR. The high prevalence of risk factors such as hypertension (70%) and smoking (83%) among our patients mirrors the risk profiles seen nationally.

Post-EVAR endoleak complications were meticulously monitored. The number of endoleaks may depend on the time elapsed since the procedure [8] and the sensitivity of the follow-up scan used [9]. Primary endoleaks were identified in the first surveillance scan before discharge, with several cases requiring further intervention, such as cuff extensions and stent placements, often in consultation with specialized centers. Secondary endoleaks, noted in 5.94% of patients during subsequent follow-ups, were predominantly type 2, highlighting the need for ongoing surveillance post-EVAR. These rates are comparable to national figures, emphasizing the critical role of follow-up in managing potential complications. 

Reduced intensive care unit and hospital length of stay are advantages of EVAR compared to open aneurysm repair [10]. The analysis of hospital stays post-EVAR in our institution revealed a mean stay of 4.21 days in 2020, with a median of three days, and notable outliers due to specific complications. In 2021, the median stay decreased to two days, with a mean of 3.11 days, reflecting improvements in perioperative care. Nationally, the NVR data also show similar trends towards reduced hospital stays, indicating better management practices and enhanced recovery protocols. Our institution's 30-day post-EVAR mortality rate was 0.9%, with one death recorded in 2016. This rate aligns closely with national statistics, underscoring the generally safe profile of EVAR within the early postoperative period. However, the lack of detailed electronic discharge documentation in this case highlights the need for comprehensive record-keeping to better understand and address mortality causes.

Post-EVAR morbidity was tracked up to six weeks after the procedure, revealing complications such as chest infections (three cases), wound infections (two cases), and one seroma. These findings underscore the importance of vigilant postoperative care, a concern reflected in national data, which also emphasise the need for robust follow-up to manage such morbidities effectively.

Given the increasing prevalence of EVAR and its reduced complication rates, this study aims to evaluate our institution’s EVAR outcomes compared to national benchmarks. Our results align with existing literature, reinforcing the feasibility of delivering EVAR services in district general hospitals. The variety of devices used in our EVAR procedures included Cook Medical (63.9%), Cordis (21.3%), Gore (6.5%), and Anaconda (3.7%). This distribution is indicative of both the institutional preferences and the anatomical considerations dictating device selection. Nationally, the NVR data reflect similar trends in device utilisation, with Cook Medical devices being widely favoured for their reliability and suitability in diverse clinical scenarios. Overall, our institution's EVAR outcomes and practices align well with national trends, demonstrating our commitment to high standards of care and continuous improvement in managing AAA.

Limitations 

This retrospective study lacks a control group, limiting causal inference. Additionally, patients treated outside the instructions for use (IFU) for specific stent grafts were not analyzed separately, which could influence complication rates, including endoleaks. The other important issue is that we did not separate patients treated within and without IFU for the specific stent grafts, which may influence the outcome including the number of endoleaks. The study’s retrospective design may introduce selection bias, as only patients with complete data were included. The results may not be generalizable to larger centers or different patient populations due to the single-center nature of the study. We acknowledge that some patients with incomplete follow-up data were excluded, which could skew the complication and survival rates reported.

## Conclusions

Finally, this seven years of experience with EVAR in a district-level hospital has shown the benefit of this procedure as a minimally invasive surgery in the elective setting. The outcome was comparable to the national standard. The complications, hospital stay, secondary interventions, and survival rates are in concordance with the national standard. This study provided a detailed account of the successful implementation and performance of the EVAR programme at a vascular centre in a district hospital. Our study has identified the strengths and areas for improvement in our EVAR practices at the district level. This provides recommendations for future practice and potential areas for research and development in EVAR.

## References

[1] Patel R, Powell JT, Sweeting MJ, Epstein DM, Barrett JK, Greenhalgh RM (2018). The UK endovascular aneurysm repair (EVAR) randomised controlled trials: long-term follow-up and cost-effectiveness analysis. Health Technol Assess.

[2] Aggarwal S, Qamar A, Sharma V, Sharma A (2011). Abdominal aortic aneurysm: a comprehensive review. Exp Clin Cardiol.

[3] Huang X, Wang Z, Shen Z (2022). Projection of global burden and risk factors for aortic aneurysm - timely warning for greater emphasis on managing blood pressure. Ann Med.

[4] Varabyova Y, Blankart CR, Schreyögg J (2017). The role of learning in health technology assessments: an empirical assessment of endovascular aneurysm repairs in German hospitals. Health Econ.

[5] Greenhalgh RM, Brown LC, Powell JT, Thompson SG, Epstein D, Sculpher MJ (2010). Endovascular versus open repair of abdominal aortic aneurysm. N Engl J Med.

[6] Qin C, Chen L, Xiao YB (2014). Emergent endovascular vs. open surgery repair for ruptured abdominal aortic aneurysms: a meta-analysis. PLoS One.

[7] Waton S, Johal A, Heikkila K, Cromwell D, Boyle J, Miller F (2024). National Vascular Registry: state of the nation report 2023. National Vascular Registry.

[8] Charisis N, Bouris V, Conway AM, Labropoulos N (2021). A systematic review and pooled meta-analysis on the incidence and temporal occurrence of type II endoleak following an abdominal aortic aneurysm repair. Ann Vasc Surg.

[9] Mirza TA, Karthikesalingam A, Jackson D, Walsh SR, Holt PJ, Hayes PD, Boyle JR (2010). Duplex ultrasound and contrast-enhanced ultrasound versus computed tomography for the detection of endoleak after EVAR: systematic review and bivariate meta-analysis. Eur J Vasc Endovasc Surg.

[10] Gavali H, Mani K, Tegler G, Kawati R, Covaciu L, Wanhainen A (2017). Editor's choice - prolonged ICU length of stay after AAA repair: analysis of time trends and long-term outcome. Eur J Vasc Endovasc Surg.

